# Sex differences in membrane properties and cellular excitability of dopamine D1 receptor-expressing neurons within the shell of the nucleus accumbens of pre- and mid-adolescent mice

**DOI:** 10.1186/s13293-024-00631-1

**Published:** 2024-07-13

**Authors:** Heather C. Aziz, Regina A. Mangieri

**Affiliations:** Division of Pharmacology and Toxicology, College of Pharmacy, The University of Texas at Austin, 2409 University Avenue, Austin, TX 78712 USA

**Keywords:** Glutamate, Electrophysiology, Synapse, Excitability, Sex differences, Striatum, Development, Ontogeny

## Abstract

**Background:**

The transition from childhood to adulthood, or adolescence, a developmental stage, is characterized by psychosocial and biological changes. The nucleus accumbens (NAc), a striatal brain region composed of the core (NAcC) and shell (NAcSh), has been linked to risk-taking behavior and implicated in reward seeking and evaluation. Most neurons in the NAc are medium spiny neurons (MSNs) that express dopamine D1 receptors (D1R +) and/or dopamine D2 receptors (D2R +). Changes in dopaminergic and glutamatergic systems occur during adolescence and converge in the NAc. While there are previous investigations into sex differences in membrane excitability and synaptic glutamate transmission in both subdivisions of the NAc, to our knowledge, none have specified NAcSh D1R + MSNs from mice during pre- and mid-adolescence.

**Methods:**

Sagittal brain slices containing the NAc were prepared from B6.Cg-Tg(Drd1a*-*tdTomato)6Calak/J mice of both sexes from postnatal days 21–25 and 35–47, representing pre- and mid-adolescence, respectively. Whole-cell electrophysiology recordings were collected from NAcSh D1R + MSNs in the form of membrane-voltage responses to current injections, to assess membrane properties and action potential waveform characteristics, and spontaneous excitatory postsynaptic currents (sEPSCs) to assess glutamatergic synaptic activity.

**Results:**

Relative to pre-adolescent males, pre-adolescent female NAcSh D1R + MSNs exhibited a less hyperpolarized resting membrane potential, increased input resistance, and smaller action potential afterhyperpolarization amplitudes. During mid-adolescence, decreased input resistance and a shorter action potential duration in females were the only sex differences observed.

**Conclusions:**

Taken together, our results indicate that NAcSh D1R + MSNs in mice exhibit sex differences in membrane properties and AP waveform during pre-adolescence that are overall indicative of increased cellular excitability in females and are suggestive of possible sex differences in glycine receptors, inwardly-rectifying potassium channels, and large conductance voltage-gated potassium channels. These differences do not appear to persist into mid-adolescence, when sex was observed to affect input resistance oppositely to that of pre-adolescence and AP waveform in a manner suggestive of differences in voltage-gated potassium channels.

## Plain language summary

Adolescence marks a period of substantial changes in both the mind and body, where alterations in the brain’s structure can influence behavior. One change in behavior exhibited by many adolescents is an increased tendency to take risks, particularly in males. While taking risks can result in positive outcomes, like learning new skills, it can also lead to reckless behaviors that may result in negative outcomes. The nucleus accumbens, a brain region tied to risk-taking and reward perception, is not well-studied during the transition from childhood to adulthood, particularly in terms of sex differences. To fill this gap in understanding, this study examined a specific type of brain cell in the nucleus accumbens of pre- and mid-adolescent male and female mice. We measured the electrical properties of these cells and assessed how they responded to manipulation of their electrical state. We also measured how much and how often excitatory electrical information is sent to these cells from other brain regions. Our results suggest that in pre-adolescent females, these brain cells are more excited by manipulations of their electrical state and that these brain cells in mid-adolescent males may take longer to communicate information to other brain regions than in similarly aged females. Understanding these intricacies of brain cell communication sheds light on potential sex-specific vulnerabilities during the transition from childhood to adulthood.

## Highlights

- Sex, as a biological variable, impacts measures of membrane properties and cellular excitability of dopamine D1 receptor-expressing medium spiny neurons within the shell of the nucleus accumbens during pre- and mid-adolescence.
- Developmental stage impacts the effects sex has on membrane properties and excitability.
- Sex, as a biological variable, does not impact basal glutamate transmission during pre- or mid-adolescence.
- This study is one of the first to assess sex-differences in this cell type in this brain region during pre- and mid-adolescence.

## Introduction

Adolescence is a stage of development characterized by psychosocial and biological changes that can contribute to an increased propensity to take risks [1, 2]. This tendency toward risk-taking, or choosing an action for which the outcome is more variable, is greater during puberty than childhood and increases across adolescence [3, 4]. During development, risk-taking plays an important role in one’s ability to define oneself and learn new skills, but it can also lead to reckless behavior [1, 5]. Across the span of adolescence, developmental alterations in the brain are widespread, multifaceted, and work to shape the childhood brain into that of adulthood.

One brain structure that is heavily modified during adolescence and linked to risk-taking behavior is the nucleus accumbens (NAc), found within the ventral striatum. Implicated in the seeking and evaluation of reward [6, 7], the NAc has two subregions: the shell (NAcSh) and the core (NAcC). Both subregions contain dopamine (DA) receptor and glutamate receptor-expressing, ƴ-aminobutyric acid-producing (GABAergic) medium spiny neurons (MSNs). NAcSh MSNs project to brain structures mediating decision making, rewarding properties of substances, and reinforcing properties of novelty, and NAcC MSNs innervate structures associated with impulsive choices, spatial learning, and responses to motivational stimuli [8–10]. MSNs have been classified by the subtype of DA receptor that they express: dopamine D1 receptor-expressing (D1R +) or dopamine D2 receptor-expressing (D2R +) MSNs. Both subtypes receive synaptic excitation via glutamatergic inputs from several forebrain structures, and dopaminergic (DAergic) inputs from the midbrain serve to modulate MSN membrane properties and excitability [9, 11].

During adolescence, drastic changes in both the DAergic and glutamatergic systems occur [12, 13], some of which appear to be sex specific. Striatal increases in DA concentration, transporters, receptor binding, and receptor expression have been documented as occurring during adolescence [1, 14–17]. Overall, the ratio of D1R + to D2R + neurons in the ventral striatum increases during adolescence, with female mice exhibiting a heightened ratio compared to males [18]. Male D1R expression and binding peaks during adolescence, whereas female D1R expression appears to peak prior to adolescence, and although striatal D1R pruning occurs in both sexes, it occurs at different ages and via different mechanisms in males and females [19, 20]. Regarding glutamate, both the frequency and amplitude of spontaneous excitatory postsynaptic currents in NAc MSNs of male rats were shown to decline during adolescence [21]. Therefore, it is likely that both DAergic and glutamatergic actions in the NAc play a role in the transition from childhood to adulthood.

Although previous bodies of work have investigated whether MSNs in the NAc exhibit sex-related differences during adolescence [22–24], none of these studies addressed mid-adolescence directly or specifically evaluated D1R + MSNs in the NAcSh of mice. Considering the evidence for DAergic and glutamatergic changes in the striatum during adolescence, that DA modulates MSN excitability, and that glutamate functions as a major excitatory neurotransmitter in the brain, we examined membrane excitability and synaptic glutamate transmission in NAcSh D1R + MSNs from gonadally intact mid-adolescent mice. We then performed a follow-up study in pre-adolescent mice, to determine if any observed sex differences in NAcSh D1R + MSNs remained consistent between these developmental stages.

## Methods

### Subjects

All whole-cell electrophysiology recordings were conducted in brain slices prepared from male and female bacterial artificial chromosome transgenic B6.Cg-Tg(Drd1a*-*tdTomato)6Calak/J mice (developed by Ade and colleagues [25] (RRID:IMSR_JAX:016204)) from a colony maintained in our laboratory. Animals were group-housed on a 12:12 reverse light cycle (ZT0 = 21:30) in standard mouse cages containing wood chip bedding (Sani-Chips; PJ Murphy). The animal housing room contained both sexes. Animals were provided with enrichment in the form of compressed cotton fiber squares (Nestlets; Ancare). Food (Prolab®5LL2 RMH 1800; LabDiet) and water were supplied ad libitum. Mice used during pre-adolescence were not weaned from their dam prior to experiments. Mice used during mid-adolescence were weaned at PND 21. All mice were ear punched for identification and genotyping at PND 14 and PND 21 for pre-adolescence and mid-adolescence, respectively. On the day of the experiment, mice ranged in age from PND 21-PND 25, representing pre-adolescence, and PND 35-PND 47, representing mid-adolescence [26]. Animal numbers for pre-adolescent males, pre-adolescent females, mid-adolescent males, and mid adolescent females were 14, 12, 40, and 46, respectively.

All animal procedures were performed in accordance with the University of Texas at Austin’s institutional animal care and use committee’s regulations.

### Brain slice preparation

Animals were lightly anesthetized with isoflurane prior to killing via decapitation. Decapitation took place between 07:00 and 13:30 (ZT9.5 and ZT16). Immediately following removal, the brains were rapidly cooled in ice-cold artificial cerebral spinal fluid (ACSF), continuously bubbled with 95% O_2_/5% CO_2_, and composed of (in mM) 210 sucrose, 26.2 NaHCO_3_, 1 NaH_2_PO_4_, 2.5 KCl, 11 glucose, 6 MgSO_4_, and 2.5 CaCl_2_. Using a vibratome (VT-100S; Leica), 240 µM thick sagittal slices containing the nucleus accumbens were made. Each slice was subsequently transferred to a recovery chamber containing ACSF constantly bubbled with 95% O_2_/5% CO_2_ and composed of (in mM): 124 NaCl, 26 NaHCO_3_, 10 dextrose, 4.4 KCl, 1 NaH_2_PO_4_, 2.4 MgSO_4_, and 1.8 CaCl_2_. Slices were incubated at 32 °C in the recovery chamber for a minimum of 60 min prior to being transferred to the recording chamber.

The recording chamber contained ACSF composed of (in mM): 124 NaCl, 26 NaHCO_3_, 1 dextrose, 4.4 KCl, 1 NaH_2_PO_3_, 1.2 MgSO_4_, and 2.0 CaCl_2_. To block ƴ-aminobutyric acid subtype A (GABA_A_) receptor-mediated currents, recording ACSF also included 50 µM picrotoxin. To validate α-amino-3-hydroxy-5-methyl-4-isoxazolepropionic acid (AMPA) and kainate receptor activity, a subset of experiments were conducted in which the recording ACSF also included the AMPA/kainate receptor antagonist DNQX (20 µM). ACSF was maintained at a temperature ranging from 32 to 33 °C and pumped continuously into the recording chamber at a rate of ~ 2 mL/min. Whole-cell recordings were made using electrodes with resistances between 3 and 7 MΩ. Electrodes were fabricated from 4″ thin-walled glass capillaries (1.5 OD/1.12 ID; World Precision Instruments) using a Flaming/Brown micropipette puller (P-97; Sutter Instruments). Recording electrodes were filled with an intracellular solution composed of (in mM): 135 KMeSO_4_, 12 NaCl, 10 HEPES, 0.5 EGTA, 2 Mg^2+^-ATP, and 0.3 Tris-GTP. All chemical components were obtained from either Sigma‒Aldrich or Fisher Scientific.

### Data acquisition and analysis

**Fig. 1:**
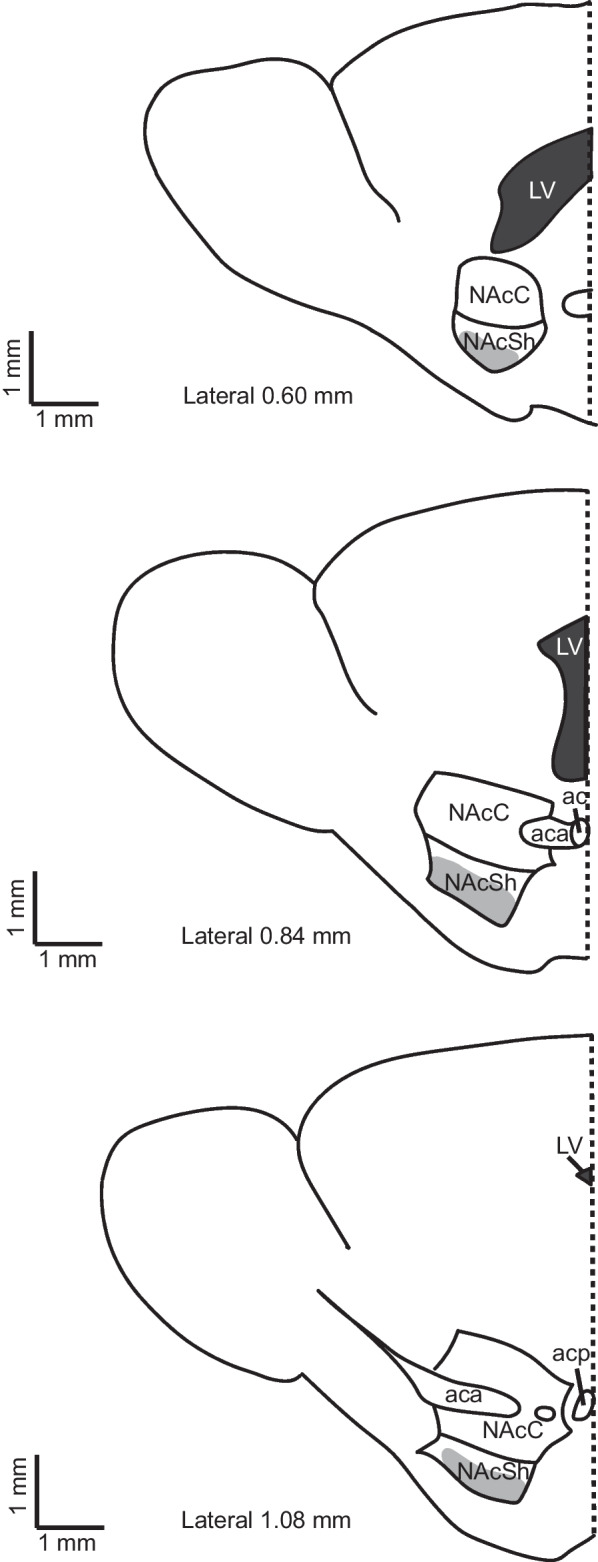
Representative illustrations of the NAc within the sagittal brain sections used for experiments. Gray shading indicates the area from which cells were selected for recordings. Anatomical landmarks, such as the anterior commissure, were used to determine which slices contained medial shell. Lateral indications (based on the adult mouse brain atlas) for each slice illustration are provided to serve as a guide for where those landmarks would be observed in an adult. The dotted line indicates Bregma. anterior commissure (ac), anterior part of the anterior commissure (aca), posterior part of the anterior commissure (acp), lateral ventricle (LV), nucleus accumbens core (NAcC), nucleus accumbens shell (NAcSh)

Whole-cell recordings were collected from D1R + MSNs located in the NAcSh (Fig. 1). Cells were identified as D1R + by the presence of epifluorescent illumination of tdTomato using the MRK200 Modular Imaging system (Siskiyou Corporation). Data was acquired utilizing a CV203BU headstage mounted on a vibration isolation table and an Axopatch 200B amplifier, with 1 kHz filtering. Data was digitized at 5 kHz through a Digidata 1440A interface board using Clampex 10.3 (all products by Molecular Devices, Sunnyvale, CA, United States). Immediately after obtaining whole-cell configuration, cells were selected for further experimentation by exhibiting a series resistance of less than 33 MΩ and having a resting membrane potential of less than or equal to -60 mV. Any electrophysiology recording during which series resistance changed by more than 20% or exceeded 33 MΩ was excluded from statistical analysis. Thus, although the general procedure was to perform a current clamp recording followed by a voltage clamp recording in the same neuron, not all neurons contributed to the final datasets for each type of experiment. Raw data analysis was performed in Clampfit 10.6 (Molecular Devices). Data analysis took place after all data was collected and was performed blind to sex.

Membrane voltage responses were measured in current clamp mode by recording 20 sweeps 750 ms in duration that included in order: 50 ms of no current application, 150 ms of − 20 pA, 150 ms of no current application, 300 ms of either a hyperpolarizing or depolarizing current, and 100 ms of no current application. The 300 ms hyperpolarizing or depolarizing current steps increased in 50 pA increments from − 400 to 550 pA. The resting membrane potential was defined as the average voltage (mV) during the initial 50 ms of the 0 pA sweep. The steady state of the voltage response to the 150 ms − 20 pA step was used to calculate the input resistance near resting membrane potential. The steady state of the voltage response to the 300 ms steps was used to determine the I-V relationship and to calculate the input resistance over a range of membrane potentials. Rheobase was defined as the amplitude of the current step that elicited the first action potential (AP) for each cell. AP waveforms were analyzed using the first AP in the rheobase step, unless the AP fired too close to the end of the step to measure AHPs; in these cases, the first AP of the next step was used. The AP threshold was defined as the point at which dV/dt exceeded 10 mV/ms for the first AP. AP amplitude was calculated by subtracting the AP threshold from the AP peak voltage. AP half-width was defined as the duration between the AP threshold and the AP half-amplitude. AP afterhyperpolarization potential (AHP) amplitudes were defined as the difference between the threshold voltage and the most negative voltage within 5 ms of threshold, the voltages at 10 and 15 ms after the AP threshold, and the most negative voltage reached during any phase of afterhyperpolarization, for fast (fAHP), medium (mAHP), slow (sAHP), and maximum (maxAHP), respectively [27, 28]. The overall maximum peak to peak frequency (Hz) was determined from the shortest interval between any two AP spikes across all current steps. The maximum peak to peak frequency (Hz) for each current step was determined from the shortest interval between any two AP spikes. Initial and steady state interspike intervals (ISIs) were defined as the time between the first and second AP spikes, and the last two AP spikes, respectively, for each current step that evoked at least 3 APs. The sweep with the maximum number of APs was used to determine the spike frequency adaptation ratio (SFA). SFA was calculated by dividing the initial ISI by the steady state ISI.

Spontaneous excitatory postsynaptic currents (sEPSCs) were monitored in voltage clamp mode, with a command potential of − 80 mV. The sEPSC frequency and average amplitude were determined over a 1–3-min period by utilizing the Clampfit template search feature and rejecting events smaller than 5 pA and with a template match less than 4 or an area smaller than − 45 pA*ms. To validate AMPA and kainate receptor activity, sEPSC amplitude and frequency were determined over a 1-min period after the brain slice was exposed to ACSF containing 20 µM DNQX for a minimum of 6 min. Following application of DNQX, we observed a 98–100% reduction in the frequency of sEPSCs (n’s (cells/mice) = (2/2) pre-adolescent males, (3/3) pre-adolescent females, (2/2) mid-adolescent males, and (2/2) mid-adolescent females), indicating that essentially all sEPSCs were AMPA- or kainate receptor-mediated.

**Fig. 2:**
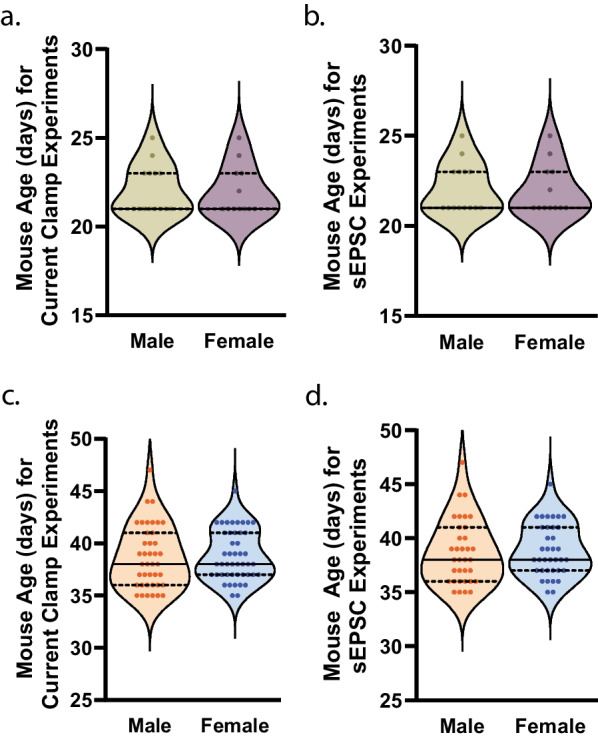
Age on experimental day. **a** Age of pre-adolescent male (green circles; n = 14) mice and female (purple circles; n = 12) mice, from which current clamp recordings were collected, on the day of the experiment. Individual values for each mouse are plotted and presented alongside a violin plot that includes the group median (solid line) as well as the upper and lower quartiles (dashed lines). **b** Age of pre-adolescent male (green circles; n = 14) mice and female (purple circles; n = 12) mice, from which spontaneous excitatory postsynaptic current (sEPSC) recordings were collected, on the day of the experiment. Individual values for each mouse are plotted and presented alongside a violin plot that includes the group median (solid line) as well as the upper and lower quartiles (dashed lines). **c** Age of mid-adolescent male (orange circles; n = 40) mice and female (blue circles; n = 46) mice, from which current clamp recordings were collected, on the day of the experiment. Individual values for each mouse are plotted and presented alongside a violin plot that includes the group median (solid line) as well as the upper and lower quartiles (dashed lines). **d** Age of mid-adolescent male (orange circles; n = 34) mice and female (blue circles; n = 36) mice, from which sEPSC recordings were collected, on the day of the experiment. Individual values for each mouse are plotted and presented alongside a violin plot that includes the group median (solid line) as well as the upper and lower quartiles (dashed lines)

Membrane voltage response experiments were taken in cells from pre-adolescent male (106/14) and female (106/12) mice, each sex having a mean age of PND 22, at death (t(24) = 0.1298, p = 0.8978)^a^ (Fig. 2a). For sEPSC experiments in pre-adolescent animals, cells were from male (70/14) and female (78/12) mice, each sex having a mean age of PND 22, at death (t(24) = 0.1298, p = 0.8978)^b^ (Fig. 2b). Membrane voltage response experiments were taken in cells from mid-adolescent male (96/40) and female (120/46) mice, each sex having a mean age of PND 39, at death (t(84) = 0.2235, p = 0.8237)^c^ (Fig. 2c). For sEPSC experiments in mid-adolescent animals, cells were from male (58/34) and female (67/36) mice, each sex having a mean age of PND 39, at death (t(68) = 0.2391, p = 0.8118)^d^ (Fig. 2d).

### Statistics

Statistical analyses were performed in GraphPad Prism version 9.3 or higher. Pre- and mid-adolescent datasets were not compared to each other due to differences in timing of data collection and to avoid impact of seasonality. For pre-adolescence, electrophysiology data was collected over the span of 5 weeks. For mid-adolescence, data was collected over the period of seven years and throughout all seasons. The current step-evoked action potential number and change in membrane voltage, as well as input resistance across current step amplitudes were analyzed by two-way ANOVA (sphericity not assumed), with sex as a between-group factor and current step as a repeated measure, followed by Sidak’s multiple comparisons of sex at each current step. For maximum peak to peak frequency and initial and steady state ISIs across current steps, mixed-effects analyses were conducted, followed by Sidak’s multiple comparisons of sex at each current step. Cumulative probability distributions of sEPSC amplitudes and interevent intervals were analyzed by two-way ANOVA (sphericity not assumed), with sex as a between-group factor and bin as a repeated measure, followed by Sidak’s multiple comparisons of sex at each bin. The effect of sex on all other variables was analyzed using unpaired Student’s *t* tests; Welch’s correction was applied when homogeneity of variance was violated. Within each age group, within-sex Pearson correlation coefficients were used to assess whether postnatal day correlated with electrophysiological measures. Tables 1 and 2 show details of the statistical analysis for pre-adolescence and mid-adolescence, respectively. Superscripts accompany reported statistics within the results for ease of reference to line in table; a-ag being included in Table 1 and ah-bn being included in Table 2.

**Table 1:** Statistical table for animal ages at death and electrophysiology measures collected during pre-adolescence

Manuscript reference	Type of test	95% Confidence interval	
a	Student's *t* test	Male	21.27 to 22.87
		Female	21.10 to 22.90
b	Student's *t* test	Male	21.27 to 22.87
		Female	21.10 to 22.90
c	Student's *t* test	Male	37.72 to 39.63
		Female	38.10 to 39.51
d	Student's *t* test	Male	37.66 to 39.75
		Female	38.04 to 39.68
e	Student's *t* test	Male	− 77.10 to − 75.83
		Female	− 76.15 to − 74.76
f	Student's *t* test	Male	85.91 to 99.44
		Female	93.63 to 109.50
g	Two-way ANOVA	Male	55.21 to 84.64
		Female	59.39 to 95.22
h	Two-way ANOVA	Male	− 16.25 to − 3.981
		Female	− 17.68 to − 4.270
i	Student's *t* test	Male	316.9 to 352.7
		Female	285.6 to 328.5
j	Student's *t* test	Male	137.7 to 163.0
		Female	140.3 to 165.5
k	Student's *t* test	Male	− 30.09 to − 28.45
		Female	− 30.42 to − 28.83
l	Student's *t* test	Male	69.38 to 72.97
		Female	69.76 to 73.25
m	Student's *t* test	Male	1.186 to 1.262
		Female	1.212 to 1.280
n	Student's *t* test	Male	4.393 to 6.293
		Female	5.240 to 7.364
o	Student's *t* test	Male	− 17.51 to − 16.18
		Female	− 16.12 to − 14.88
p	Student's *t* test	Male	− 17.09 to − 15.66
		Female	− 15.60 to − 14.22
q	Student's *t* test	Male	− 14.21 to − 13.18
		Female	− 13.04 to − 11.99
r	Student's *t* test	Male	− 13.38 to − 12.21
		Female	− 12.31 to − 11.14
s	Two-way ANOVA	Male	1.646 to 7.255
		Female	2.350 to 6.937
t	Welch's *t* test	Male	55.49 to 63.79
		Female	56.59 to 69.10
u	Mixed-effects analysis	Male	31.43 to 48.52
		Female	31.26 to 54.11
v	Mixed-effects analysis	Male	25.10 to 37.50
		Female	23.81 to 42.21
w	Mixed-effects analysis	Male	36.98 to 52.63
		Female	36.02 to 54.40
x	Student's *t* test	Male	0.6107 to 0.6790
		Female	0.6235 to 0.6955
y	Student's *t* test	Male	− 23.65 to − 22.25
		Female	− 23.61 to − 22.16
z	Two-way ANOVA	Male	0.9318 to 0.9866
		Female	0.9321 to 0.9868
aa	Student's *t* test	Male	3.340 to 4.741
		Female	3.007 to 4.416
ab	Two-way ANOVA	Male	0.9223 to 0.9757
		Female	0.9129 to 0.9703
ac	Pearson correlation	Male	0.037 to 0.400
ad	Pearson correlation	Male	− 0.424 to − 0.048
ae	Pearson correlation	Female	0.116 to 0.464
af	Pearson correlation	Female	0.030 to 0.394
ag	Pearson correlation	Female	− 0.371 to − 0.004

**Table 2:** Statistical table for measures collected during mid-adolescence

Manuscript reference	Type of test	95% Confidence interval	
ah	Student's *t* test	Male	− 71.27 to − 69.20
		Female	− 70.45 to − 68.74
ai	Student's *t* test	Male	113.40 to 136.70
		Female	103.50 to 125.30
aj	Two-way ANOVA	Male	74.52 to 101.40
		Female	67.53 to 91.57
ak	Two-way ANOVA	Male	− 23.39 to − 8.831
		Female	− 21.22 to − 7.941
al	Student's *t* test	Male	252.90 to 290.30
		Female	264.90 to 304.90
am	Student's *t* test	Male	100.60 to 129.50
		Female	106.60 to 133.60
an	Student's *t* test	Male	− 28.25 to − 26.10
		Female	− 27.33 to − 25.24
ao	Student's *t* test	Male	60.16 to 63.97
		Female	62.10 to 65.66
ap	Student's *t* test	Male	1.415 to 1.524
		Female	1.347 to 1.434
aq	Student's *t* test	Male	4.997 to 7.213
		Female	5.210 to 7.183
ar	Student's *t* test	Male	− 14.52 to − 12.82
		Female	− 15.34 to − 13.77
as	Student's *t* test	Male	− 13.93 to − 12.19
		Female	− 14.74 to − 13.15
at	Student's *t* test	Male	− 11.48 to − 10.11
		Female	− 12.06 to − 10.78
au	Student's *t* test	Male	− 10.67 to − 9.152
		Female	− 11.17 to − 9.660
av	Two-way ANOVA	Male	1.239 to 3.445
		Female	1.441 to 3.686
aw	Student's *t* test	Male	49.20 to 58.87
		Female	54.00 to 64.46
ax	Mixed-effects analysis	Male	33.44 to 47.42
		Female	36.95 to 50.01
ay	Mixed-effects analysis	Male	24.68 to 32.90
		Female	25.93 to 33.17
az	Mixed-effects analysis	Male	34.63 to 46.25
		Female	35.23 to 41.11
ba	Welch's *t* test	Male	0.6618 to 0.7432
		Female	0.6588 to 0.8153
bb	Student's *t* test	Male	− 17.01 to − 14.59
		Female	− 16.55 to − 14.60
bc	Two-way ANOVA	Male	0.910 to 0.982
		Female	0.906 to 0.981
bd	Student's *t* test	Male	4.494 to 6.327
		Female	4.285 to 6.153
be	Two-way ANOVA	Male	0.914 to 0.996
		Female	0.913 to 0.994
bf	Pearson correlation	Male	0.072 to 0.477
bg	Pearson correlation	Male	0.043 to 0.424
bh	Pearson correlation	Male	− 0.445 to − 0.070
bi	Pearson correlation	Male	− 0.442 to − 0.066
bj	Pearson correlation	Female	0.012 to 0.383
bk	Pearson correlation	Female	− 0.355 to − 0.009
bl	Pearson correlation	Female	− 0.427 to − 0.088
bm	Pearson correlation	Female	− 0.386 to − 0.039
bn	Pearson correlation	Female	− 0.365 to − 0.014

## Results

### Pre-adolescence

**Fig. 3:**
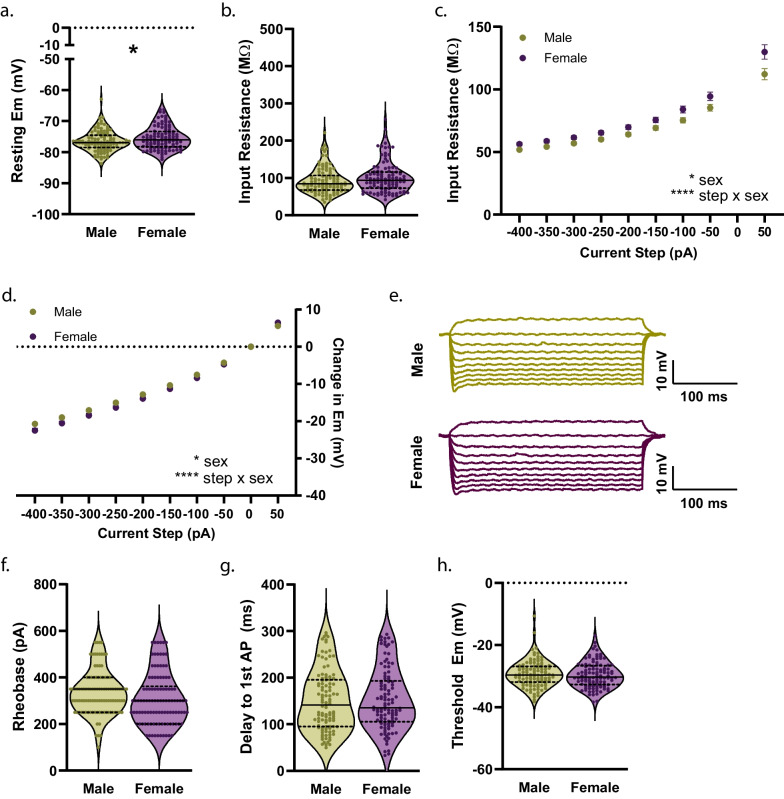
Membrane properties and cellular excitability of D1R + MSNs from male and female *Drd1a*-tdTomato mice during pre-adolescence. **a** During pre-adolescence female D1R + MSNs exhibit a more positive resting membrane potential (Em) compared to male D1R + MSNs [*p = 0.0349, unpaired t-test]. For **a**–**c**, n (cells/mice) = 106/14 male (green circles), 106/12 female (purple circles). For **a**–**b** individual values for each neuron are plotted and are presented alongside a violin plot that includes the group median (solid line) as well as the upper and lower quartiles (dashed lines). **b** Input resistance determined from a 150 ms − 20 pA current step. **c** Significant main effect of sex and [*p = 0.0156, 2way ANOVA] interaction of sex with current step amplitude on input resistance determined over a range of 300 ms current step amplitudes [****p < 0.0001, 2way ANOVA]. For c, mean and standard error of the mean are presented. **d** Significant main effect of sex [*p = 0.0180, 2way ANOVA] and interaction of sex with current step amplitude on membrane voltage change in response to application of 300 ms current steps in cells from male (green circles, n = 106/14) and female (purple circles, n = 106/12) mice [****p < 0.0001, 2way ANOVA]. For **d**, mean and standard error of the mean are presented. **e** Representative traces of voltage responses to current steps. **f** AP rheobase. For **f**–**h**, n (cells/mice) = 105/14 male (green circles), 106/12 female (purple circles); individual values for each neuron are plotted and are presented alongside a violin plot that includes the group median (solid line) as well as the upper and lower quartiles (dashed lines). **g** Delay to action potential (AP). **h** AP threshold

**Fig. 4:**
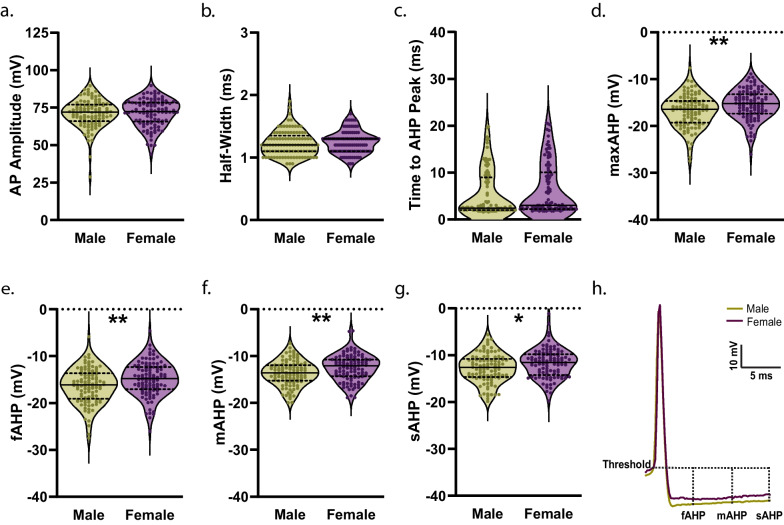
Measures of action potential (AP) waveform in D1R + MSNs from male and female mice during pre-adolescence. **a** AP amplitude. For **a**–**g**, n (cells/mice) = 105/14 male (green circles), 106/12 female (purple circles); individual values for each neuron are plotted and are presented alongside a violin plot that includes the group median (solid line) as well as the upper and lower quartiles (dashed lines). **b** AP half-width. **c** Time elapsed from threshold to afterhyperpolarization (AHP) peak. **d** D1R + MSNs from female mice exhibited a significantly smaller maximum AHP amplitude compared to males [**p = 0.0038, unpaired t-test]. **e** Fast AHP (fAHP) amplitude was significantly smaller in female D1R + MSNs compared to male during pre-adolescence [**p = 0.0039, unpaired t-test]. **f** Medium AHP (mAHP) amplitude was significantly reduced in female D1R + MSNs compared to male [**p = 0.0017, unpaired t-test]. **g** D1R + MSNs from female mice exhibited a significantly smaller slow AHP (sAHP) amplitude compared to males [*p = 0.0111, unpaired t-test]. **h** Representative traces showing AP waveform 5 ms prior to threshold through the sAHP for both male (green) and female (purple) D1R + MSNs. Traces aligned at threshold

**Fig. 5:**
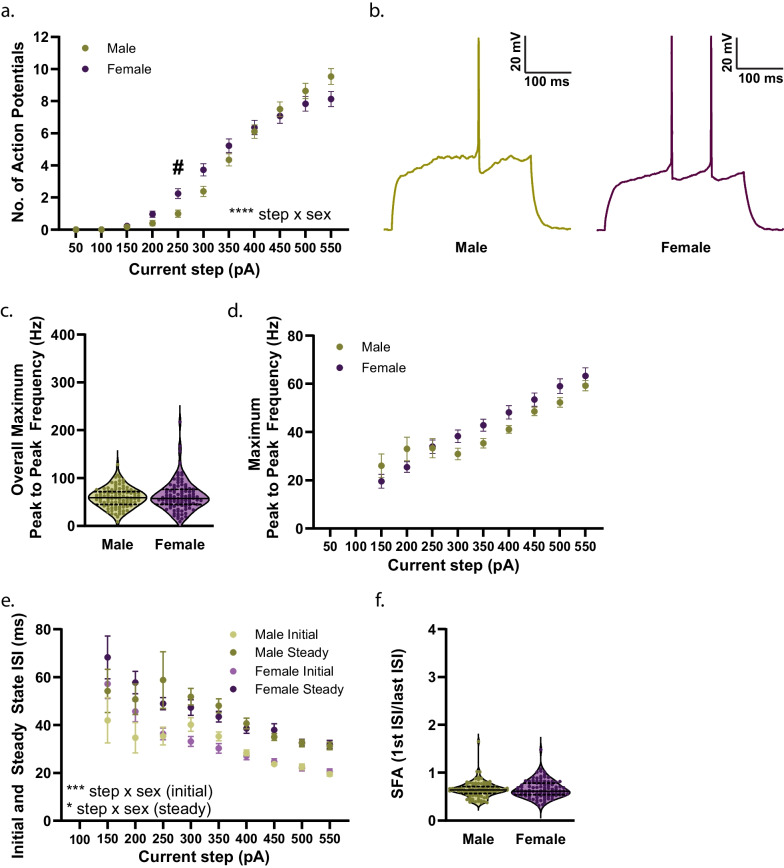
Excitability of D1R + MSNs from male and female mice during pre-adolescence. **a** Significant effect of sex on the number of action potentials (APs) elicited in response to application of depolarizing current steps (50–500 pA) [****p < 0.0001, 2way ANOVA] with a significant increase in the number of APs fired in female (purple circles) D1R + MSNs in response to a 250 pA current compared to male (green circles) D1R + MSNs [^#^*p* = 0.0100, Šídák’s t-test]. Group mean and standard error of the mean are presented. For **a**, n (cells/mice) = 106/14 male, 106/12 female. **b** Representative traces of voltage response to 250 pA current for male (green) and female (purple) D1R + MSNs during pre-adolescence. **c** Overall maximum peak to peak frequency for males (green circles) and females (purple circles). For **c**, n (cells/mice) = 100/14 male, 101/12 female; individual values for each neuron are plotted and are presented alongside a violin plot that includes the group median (solid line) as well as the upper and lower quartiles (dashed lines). **d** Maximum peak to peak AP firing frequency in response to depolarizing current steps (150–550 pA) for males (green circles) and females (purple circles). Group mean and standard error of the mean are presented. For **d**, n (cells/mice) = 100/14 male, 101/12 female. **e** Significant interaction of sex with current amplitude on initial [***p = 0.0006, mixed-effects analysis] and steady state [*p = 0.0475, 2way ANOVA] interspike intervals (ISI). For **e**, n (cells/mice) = male (94/14), female (93/12). Group mean and standard error of the mean are presented for males (green circles) and females (purple circles). **f** Spike frequency adaptation (SFA) during the current step that elicited the maximum number of APs in male (green circles; n = 97/14) and female (purple circles; n = 100/12) mice

**Fig. 6:**
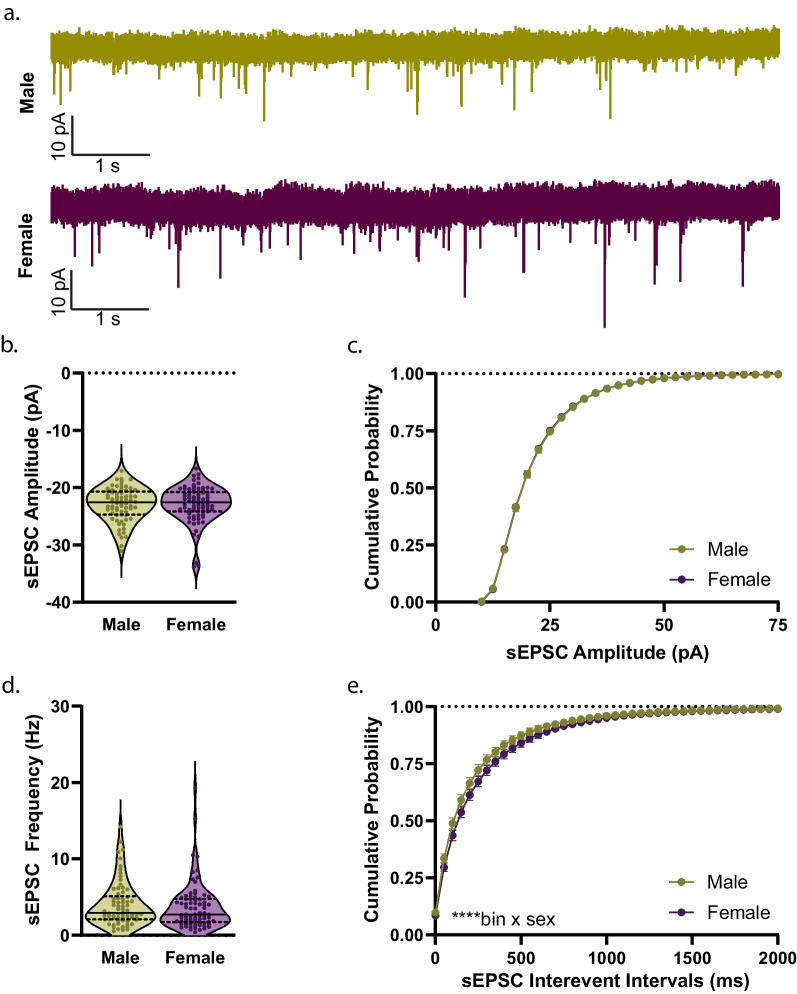
Spontaneous D1R + MSN glutamatergic transmission during pre-adolescence. **a** Example traces of spontaneous excitatory postsynaptic currents (sEPSCs) from male (green) and female mice (purple). **b** Average amplitude of sEPSCs in male (green circles) and female D1R + MSNs (purple circles). For **b**, n (cells/mice) = 70/14 male, 78/12 female; individual values for each neuron are plotted and are presented alongside a violin plot that includes the group median (solid line) as well as the upper and lower quartiles (dashed lines). **c** Cumulative frequency distribution of sEPSC event amplitudes for male (green circles) and female (purple circles). **d** Average frequency of D1R + MSN sEPSCs during pre-adolescence in males (green circles) and females (purple circles). For **d**, n (cells/mice) = 70/14 male, 78/12 female; individual values for each neuron are plotted and are presented alongside a violin plot that includes the group median (solid line) as well as the upper and lower quartiles (dashed lines). **e** Significant interaction of bin by sex for the cumulative frequency distribution of sEPSC interevent intervals for male (green circles) and female (purple circles) D1R + MSNs [****p < 0.0001, 2way ANOVA]

To establish the effect of sex on measures of membrane properties and cellular excitability in NAcSh D1R + MSNs during pre-adolescence, we collected whole-cell electrophysiology data in brain slices from male (n = 14) and female (n = 12) mice. Pre-adolescent female D1R + MSNs were observed to have a more positive resting membrane potential compared to males (*t*(210) = 2.123, *p* = 0.0349)^e^ (Fig. 3a). We found no significant effect of sex on input resistance assessed close to the resting membrane potential (input resistance determined from a 150 ms − 20 pA step) (*t*(210) = 1.689, *p* = 0.0926)^f^ (Fig. 3b). However, when examining input resistance across a range of 300 ms current steps, from − 400 to + 50 pA, we observed a significant main effect of sex (F(1, 210) = [5.939], *p* = 0.0156)^g^ and interaction of current step by sex (F(8,1680) = [4.8400], *p* < 0.0001)^g^, with input resistance being higher in females overall but not significantly different as assessed by Sidak’s multiple comparisons test at any particular current step (Fig. 3c). Correspondingly, there was a significant main effect of sex (F(1, 210) = [5.681], *p* = 0.0180)^h^ and current step by sex interaction for membrane voltage responses to current steps (F(9, 1890) = [5.930], *p* < 0.0001)^h^, and post hoc comparisons did not reveal a significant effect of sex at any particular current step (Fig. 3d, e). There was a clear trend for rheobase to be lower in females, but this was not at the level of statistical significance (*t*(209) = 1.963, *p* = 0.0510)^i^; delay to the first action potential (AP) (*t*(209) = 0.2872, *p* = 0.7743)^j^ and threshold (*t*(209) = 0.6176, *p* = 0.5375)^k^ did not differ by sex during pre-adolescence (Fig. 3f–h). No significant effect of sex was observed for AP amplitude (*t*(209) = 0.2615, *p* = 0.7940)^l^, AP half-width (*t*(209) = 0.8679, *p* = 0.3864)^m^, and time elapsed from threshold to afterhyperpolarization (AHP) peak (*t*(209) = 1.334, *p* = 0.1836)^n^ (Fig. 4a–c). We observed a significant reduction in the amplitude of the maximum AHP (*t*(209) = 2.929, *p* = 0.0038)^o^ (Fig. 4d), fast AHP (*t*(209) = 2.923, *p* = 0.0039)^p^ (Fig. 4e), medium AHP (*t*(209) = 3.186, *p* = 0.0017)^q^ (Fig. 4f), and slow AHP (*t*(209) = 2.561, *p* = 0.0111)^r^ (Fig. 4g) in female D1R + MSNs compared to males during pre-adolescence (illustrated in Fig. 4h). There was no main effect of sex on the number APs elicited in response to depolarizing current steps (F(1, 210) = [0.2482], *p* = 0.6189)^s^, but a significant current step by sex interaction was observed (F(8, 1680) = [6.258], *p* < 0.0001)^s^ with a significant increase in the number of APs elicited in response to a 250 pA current for females (Sidak’s t(195.3) = 3.309, *p* = 0.0100)^s^ (Fig. 5a, b). We found no significant effect of sex on the overall maximum (regardless of the current step in which it occurred) peak to peak AP firing frequency (Welch’s t(173.4) = 0.8466, *p* = 0.3984)^t^ (Fig. 5c) or the maximum peak to peak firing frequency during each specific current step (main effect of sex: (F(1, 199) = [0.9362], *p* = 0.3344), current step by sex interaction: F(8, 806) = [0.9196], *p* = 0.4993))^u^ (Fig. 5d). When examining whether sex impacted initial and steady state interspike intervals across all depolarizing current steps, we found a significant current step by sex interaction for both initial (F(8, 795) = [3.452], p = 0.0006)^v^ and steady state (F(8, 795) = [1.970], p = 0.0475)^w^ but no significant effect of sex for initial or steady state at individual current steps (Fig. 5e). We found no significant effect of sex on spike frequency adaptation (SFA) for the current step that elicited the maximum number of APs (*t*(195) = 0.5850, *p* = 0.5593)^x^ (Fig. 5e). When examining spontaneous glutamatergic transmission during pre-adolescence (example traces shown in Fig. 6a) we found no significant effect of sex on the average spontaneous excitatory postsynaptic current (sEPSC) amplitude (*t*(146) = 0.1270, *p* = 0.8991)^y^ (Fig. 6b), the cumulative probability distribution of sEPSC amplitudes (F(126, 18,396) = [0.03688], *p* > 0.9999)^z^ (Fig. 6c), or average sEPSC frequency (*t*(146) = 0.6570, *p* = 0.5122)^aa^ (Fig. 6d). For sEPSC frequency, when examining the cumulative probability distributions of interevent intervals, we found a significant bin by sex interaction (F(100, 14,600) = [1.967], *p* < 0.0001)^ab^, but no significant effect of sex for any particular bin (Fig. 6e). Finally, we performed within-sex correlations to determine whether mouse age correlated with any of measures of membrane properties, cellular excitability and spontaneous glutamatergic transmission (Table 3). For pre-adolescent males, we found a significant positive correlation between age and time elapsed to AHP peak (*r*(104) = 0.226, *p* = 0.0200)^ac^ and a significant negative correlation between age and SFA (*r*(95) = − 0.240, *p* = 0.0160)^ad^. For pre-adolescent females we found significant positive correlations between age and resting membrane potential (*r*(104) = 0.300, *p* = 0.0018)^ae^ and rheobase (*r*(104) = 0.220, *p* = 0.0236)^af^ and a significant negative correlation between age and AP amplitude (*r*(104) = -0.195, *p* = 0.0457)^ag^.

**Table 3:** Correlation matrix for age and electrophysiology measures during pre-adolescence

	Age	
	Male	Female
Resting Em (mV)	0.069	0.300**
Input resistance (MΩ)	− 0.123	− 0.181
Rheobase (pA)	− 0.005	0.220*
Delay to first AP (ms)	0.086	0.022
Threshold (mV)	0.056	0.169
AP AMPLITUDE (mV)	− 0.053	− 0.195*
Half-Width (ms)	0.050	− 0.094
Time to AHP (ms)	0.226*	− 0.122
maxAHP (mV)	0.079	− 0.056
fAHP (mV)	0.133	− 0.067
mAHP (mV)	− 0.050	0.045
sAHP (mV)	− 0.048	0.056
Overall max peak to peak frequency (Hz)	0.041	− 0.067
SFA (1st ISI/last ISI)	− 0.245*	0.019
sEPSC amplitude (pA)	− 0.228	0.048
sEPSC frequency (Hz)	0.104	0.209

Significant correlations with age are presented in bold (*p < 0.05, **p < 0.001)

### Mid-adolescence

**Fig. 7:**
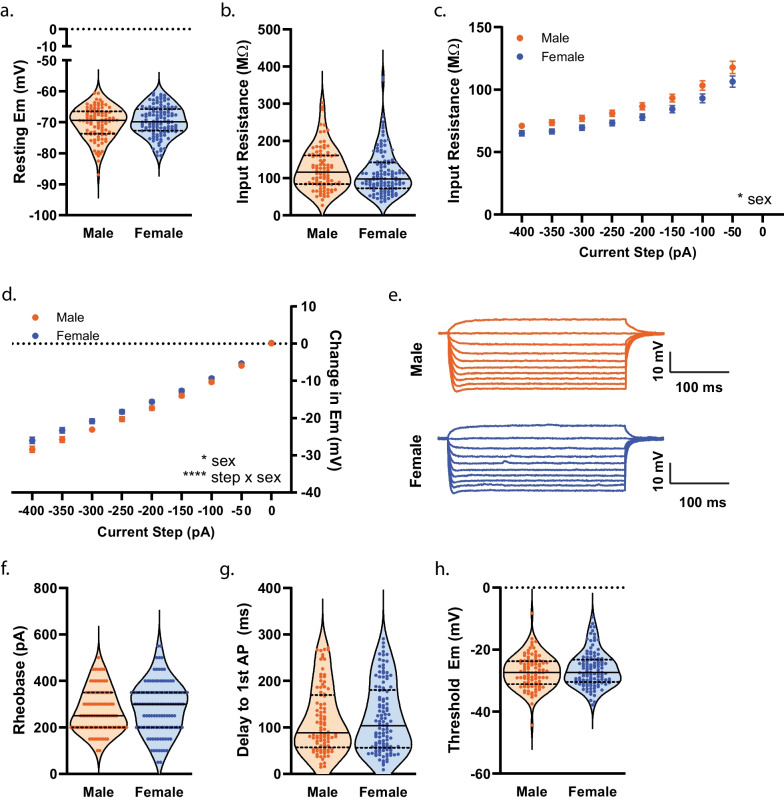
Membrane properties and cellular excitability of D1R + MSNs from male and female *Drd1a*-tdTomato mice during mid-adolescence. **a** Resting membrane potential (Em). For **a**–**d**, n (cells/mice) = 96/40 male (orange circles), 120/46 female (blue circles). For **a**–**b** individual values for each neuron are plotted and are presented alongside a violin plot that includes the group median (solid line) as well as the upper and lower quartiles (dashed lines). **b** Input resistance determined from a 150 ms − 20 pA current step. **c** Significant main effect of sex [*p = 0.0375, 2way ANOVA] on input resistance determined over a range of 300 ms hyperpolarizing current steps. For **c**–**d**, mean and standard error of the mean are presented. **d** Significant main effect of sex [*p = 0.0286, 2way ANOVA] and significant interaction of sex by step on membrane voltage change in response to application of 300 ms hyperpolarizing current steps [****p < 0.0001, 2way ANOVA]. **e** Representative traces of voltage responses to current steps. **f** AP rheobase. For **d**–**f**, n (cells/mice) = 95/39 male (orange circles), 116/46 female (blue circles); individual values for each neuron are plotted and are presented alongside a violin plot that includes the group median (solid line) as well as the upper and lower quartiles (dashed lines). **g** Delay to action potential (AP). **h** AP threshold

**Fig. 8:**
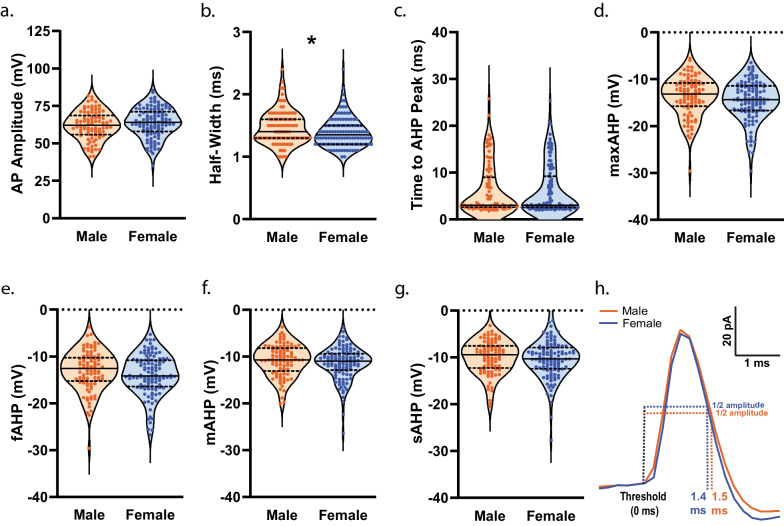
Measures of action potential (AP) waveform in D1R + MSNs from male and female mice during mid-adolescence. **a** AP amplitude. For **a**–**g**, n (cells/mice) = 95/39 male (orange circles), 116/46 female (blue circles); individual values for each neuron are plotted and are presented alongside a violin plot that includes the group median (solid line) as well as the upper and lower quartiles (dashed lines). **b** AP half-width was significantly longer in duration in D1R + MSNs from male mice than from female mice [*p = 0.0244, unpaired t-test]. **c** Time elapsed from threshold to afterhyperpolarization (AHP) peak. **d** Maximum AHP. **e** Fast AHP (fAHP). **f** Medium AHP (mAHP). **g** Slow AHP (sAHP). **h** Representative traces showing AP waveform 5 ms prior to threshold and through to 4 ms after threshold. Half-width depicted by dashed line for male (orange) and female (blue) traces as time elapsed from threshold to half amplitude. Traces aligned at threshold

**Fig. 9:**
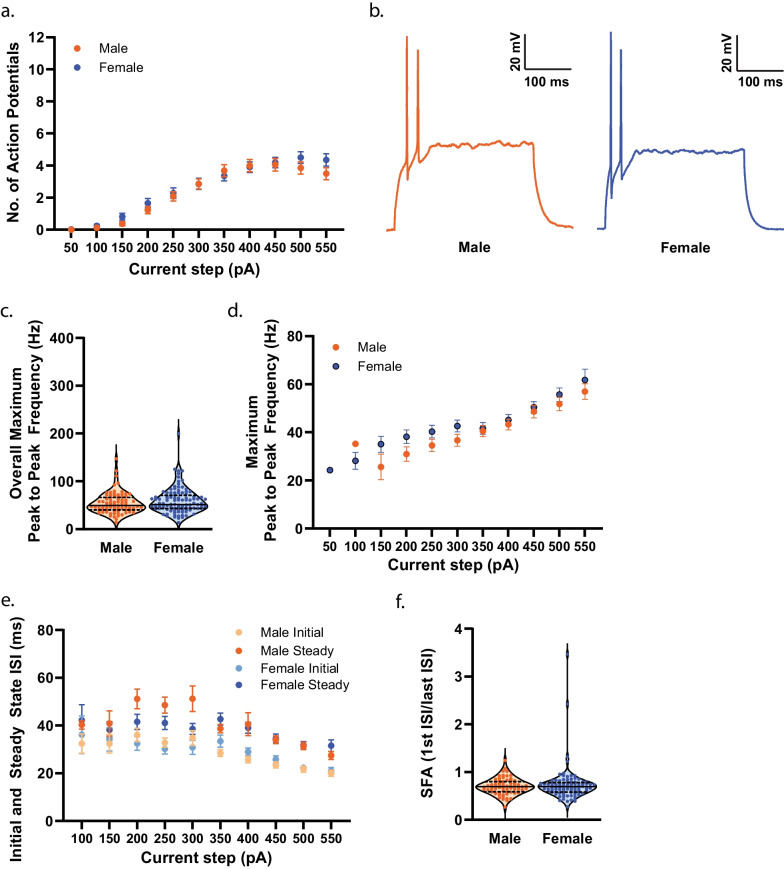
Excitability of D1R + MSNs from male and female mice during mid-adolescence. **a** Number of action potentials (APs) elicited in response to application of depolarizing current steps (50–500 pA) in male (orange circles) and female (blue circles). Group mean and standard error of the mean are presented. For **a**, n (cells/mice) = 96/40 male, 120/46 female. **b** Representative traces of voltage response to 250 pA current for male (orange) and female (blue) D1R + MSNs during mid-adolescence. ***c.*** Overall maximum peak to peak AP firing frequency for males (orange circles) and females (blue circles). For **c**, individual values for each neuron are plotted and are presented alongside a violin plot that includes the group median (solid line) as well as the upper and lower quartiles (dashed lines). For **c**–**d**, n (cells/mice) = 81/37 male, 103/45 female. **d** Maximum peak to peak AP firing frequency at each amplitude of current injected for male (orange circles) and female (blue circles) D1R + MSNs. Group mean and standard error of the mean are presented except for the 50 pA step during which only one female cell fired more than 2 APs. **e** Initial and steady state interspike intervals (ISIs) across a range of injected current amplitudes. Group mean and standard error of the mean are presented. For **e**, n (cells/mice) = 48/29 male (orange circles), 63/35 female (blue circles). **f** Spike frequency adaptation (SFA) during the current step that elicited the maximum number of APs in male (orange circles) and female (blue circles) mice. For **f**, n (cells/mice) = 68/35 male, 92/41 female; individual values for each neuron are plotted and are presented alongside a violin plot that includes the group median (solid line) as well as the upper and lower quartiles (dashed lines)

**Fig. 10:**
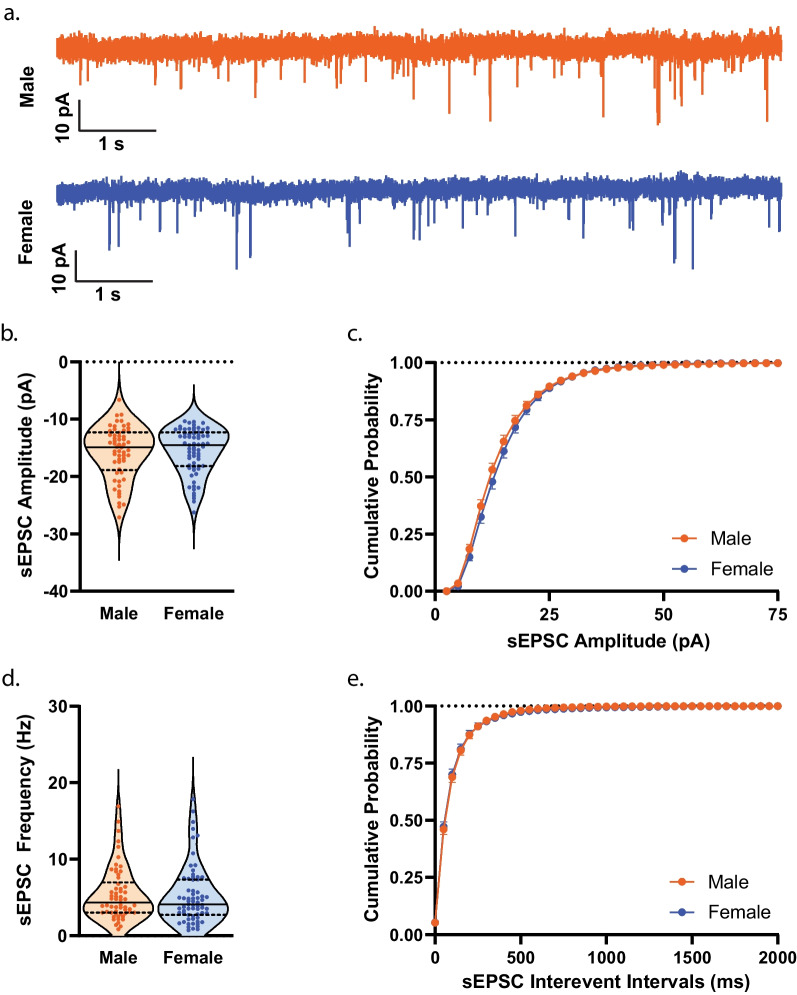
Spontaneous D1R + MSN glutamatergic transmission in male and female mice during mid-adolescence. **a** Example traces of spontaneous excitatory postsynaptic currents (sEPSCs) from male (orange) and female (blue) mice. **b** Amplitude of sEPSC in male (orange circles) and female D1R + MSNs (blue circles). For **b**, n (cells/mice) = 58/34 male, 67/36 female; individual values for each neuron are plotted and are presented alongside a violin plot that includes the group median (solid line) as well as the upper and lower quartiles (dashed lines). **c** Frequency distribution of sEPSC event amplitude for male (orange circles) and female (blue circles). **d** Frequency of D1R + MSN sEPSCs during pre-adolescence in males (orange circles) and females (blue circles). For **d**, n (cells/mice) = 58/34 male, 67/36 female; individual values for each neuron are plotted and are presented alongside a violin plot that includes the group median (solid line) as well as the upper and lower quartiles (dashed lines). **e** Frequency distribution of sEPSC interevent intervals for male (orange circles) and female (blue circles) D1R + MSNs

To establish whether measures of membrane properties and cellular excitability in NAcSh D1R + MSNs during mid-adolescence differed by sex, we collected whole-cell electrophysiology data in brain slices from male (n = 40) and female (n = 46) mice. We observed no significant effect of sex, during mid-adolescence, on resting membrane potential (*t*(214) = 0.9602, *p* = 0.3381)^ah^ (Fig. 7a) and input resistance assessed close to the resting membrane potential (input resistance determined from a 150 ms − 20 pA step) (*t*(214) = 1.317, *p* = 0.1892)^ai^ (Fig. 7b). A main effect of sex on input resistance across increasing and hyperpolarizing current injections was observed (F(1, 214) = [4.380], *p* = 0.0375)^aj^ (Fig. 7c). A significant main effect of sex (F(1, 214) = [4.859], *p* = 0.0286)^ak^ and significant current step by sex interaction was observed for voltage responses to hyperpolarizing current steps (F(8, 1712) = [4.416], *p* < 0.0001)^ak^ (Fig. 7d, e). We found no significant effect of sex on rheobase (*t*(209) = 0.9492, *p* = 0.3436)^al^, delay to first AP (*t*(209) = 0.5075, *p* = 0.6124)^am^, and threshold (*t*(209) = 1.164, *p* = 0.2459)^an^ (Fig. 7f–h). Sex did not significantly affect AP amplitude (*t*(209) = 1.374, *p* = 0.1708)^ao^ (Fig. 8a). However, we observed a significantly longer AP half-width in D1R + MSNs from male mice (*t*(209) = 2.267, *p* = 0.0244)^ap^ compared to female D1R + MSNs (Fig. 8b, h). Sex was not observed to have an effect on the time elapsed between threshold and AHP peak (*t*(209) = 0.1222, *p* = 0.9028)^aq^, maximum AHP (*t*(209) = 1.503, *p* = 0.1343)^ar^, fast AHP (t(209) = 1.487, *p* = 0.1386)^as^, medium AHP (*t*(209) = 1.319, *p* = 0.1886)^at^, and slow AHP (*t*(209) = 0.9242, *p* = 0.3565)^au^ (Fig. 8c–g. We found no significant effect of sex on the number of action potentials (APs) elicited over increasing depolarizing current steps (main effect of sex: (F(1, 214) = [0.7293], *p* = 0.3941), current step by sex interaction: (F(10, 2140) = [0.9629], *p* = 0.4740))^av^ (Fig. 9a, b), the overall maximum peak to peak AP firing frequency (regardless of the current step in which it occurred) (*t*(182) = 1.415, *p* = 0.1589)^aw^ (Fig. 9c), and the maximum peak to peak AP firing frequency during each specific current step (main effect of sex: (F(1,172) = [0.0019], *p* = 0.9645), current step by sex interaction: (F(9, 793) = [1.249], *p* = 0.2615))^ax^ (Fig. 9d). We found no significant difference in the initial (main effect of sex: (F(1, 158) = [0.5964], *p* = 0.4411), current step by sex interaction: (F(9, 547) = [1.002], *p* = 0.4370))^ay^ or steady state (main effect of sex: (F(1, 158) = [0.2112], *p* = 0.6464), current step by sex interaction: (F(9, 546) = [1.674], *p* = 0.0922))^az^ interspike interval across depolarizing current steps for D1R + MSNs from males or females during mid-adolescence (Fig. 9e). SFA for the current step that elicited the maximum number of APs was also found not to differ by sex (Welch’s *t*(133.3) = 0.7792, *p* = 0.4373)^ba^ (Fig. 9f). When examining spontaneous glutamatergic transmission, we found no significant effect of sex on average sEPSC amplitude (*t*(123) = 0.2937, *p* = 0.7695)^bb^, the cumulative probability distributions of sEPSC amplitudes (F(97, 12,707) = [1.128], *p* = 0.1850)^bc^, average sEPSC frequency (*t*(123) = 0.2904, *p* = 0.7720)^bd^ and the cumulative probability distributions of sEPSC interevent intervals (F(54, 6642) = [0.1733], *p* > 0.9999)^be^ (Fig. 10a–e). We then examined whether mouse age correlated with any measures of membrane properties, cellular excitability and spontaneous glutamatergic transmission (Table 4). For mid-adolescent males we found a significant positive correlations between age and delay to AP (*r*(93) = 0.270, *p* = 0.0082)^bf^ and between age and threshold (*r*(93) = 0.243, *p* = 0.0178)^bg^. We also observed significant negative correlations between male age and medium AHP (*r*(93) = − 0.268, *p* = 0.0088)^bh^ and between male age and slow AHP (*r*(93) = − 0.264, *p* = 0.0098)^bi^. With regard to correlations with female age and measures collected, we found a significant positive correlation of age and maximum peak to peak frequency at the current step that elicited the maximum number of action potentials (*r*(101) = 0.205, *p* = 0.0380)^bj^ and significant negative correlations of age and input resistance (*r*(118) = − 0.188, *p* = 0.0396)^bk^, age and half-width (*r*(114) = − 0.266, *p* = 0.0039)^bl^, age and time elapsed from threshold to AHP peak (*r*(114) = − 0.219, *p* = 0.0180)^bm^ and age and fast AHP (*r*(114) = − 0.196, *p* = 0.0350)^bn^.

**Table 4:** Correlation matrix for age and electrophysiology measures during mid-adolescence

	Age	
	Male	Female
Resting Em (mV)	0.086	− 0.039
Input resistance (MΩ)	0.047	− 0.189*
Rheobase (pA)	− 0.107	0.097
Delay to first AP (ms)	0.270**	− 0.120
Threshold (mV)	0.243*	− 0.024
AP amplitude (mV)	0.022	0.180
Half-width (ms)	0.026	− 0.266**
Time to AHP (ms)	− 0.019	− 0.219*
maxAHP (mV)	− 0.152	− 0.112
fAHP (mV)	− 0.140	− 0.196*
mAHP (mV)	− 0.268**	0.067
sAHP (mV)	− 0.264**	0.074
Overall max peak to peak frequency (Hz)	0.211	0.205*
SFA (1st ISI/last ISI)	− 0.810	− 0.091
sEPSC amplitude (pA)	− 0.073	0.116
sEPSC frequency (Hz)	− 0.141	− 0.020

Significant correlations with age are presented in bold (*p < 0.05, **p < 0.001)

## Discussion

Our findings demonstrate sex differences in the membrane properties and cellular excitability of D1R + MSNs in the NAcSh during both pre- and mid-adolescence, with sex differences during pre-adolescence not being observed during mid-adolescence. In summary, we found a depolarized resting membrane potential for pre-adolescent female D1R + MSNs, reduced action potential (AP) afterhyperpolarization amplitude, and an increase in cellular excitability, as measured by the number of action potentials elicited in response to a 250 pA current injection and increased input resistance, compared to pre-adolescent males. During mid-adolescence we found AP duration to be shorter, and input resistance to be greater, in females than males. To our knowledge, this is the first body of work to focus solely on D1R + MSNs in the NAcSh during pre- and mid-adolescence in mice of both sexes.

Our observation that females in pre-adolescence exhibited a less hyperpolarized resting membrane potential, compared to males, may be indicative of a sex difference at this developmental stage in the influence of the potassium leak channel, inwardly-rectifying potassium (Kir) channels, and/or the glycine receptor (GlyR) on the resting membrane potential. We also observed a significant main effect of sex on input resistance in pre- and mid-adolescence, with a different direction of effect between the two age groups. In addition to influencing the resting membrane potential, Kir channels and GlyRs are also possible contributors to differences in input resistance [29–32]. Therefore, at a minimum, our results suggest there may be sex differences in Kir and GlyR expression, composition, or function that differ by developmental stage and therefore differentially impact the resting membrane potential and/or input resistance in pre- and mid-adolescent mice.

We found that resting membrane potential was positively correlated with age in females, but not males, in our pre-adolescent age group. Functional and tonically active GlyRs are present in the NAcSh, and the expression and subunit composition of GlyRs in the NAc has been shown to be in flux over the period of PND 2 through PND 60 [33–38]. Although these developmentally-related changes in expression of GlyRs were measured in both sexes, this work did not evaluate sex as a biological variable. As such, we cannot discount the possibility that the timing of the developmental changes in GlyR subunit expression varies between males and females. Thus, the sex difference in resting membrane potential of D1R + MSNs during pre-adolescence may be a result of a sex-related difference in the ontogeny of the GlyR, either in subunit composition or expression.

The present study found a significantly reduced afterhyperpolarization (AHP) amplitude in pre-adolescent female D1R + MSNs compared to males and as measured by maximum AHP peak, fast AHP (fAHP), medium AHP (mAHP) and slow AHP (sAHP). These measures were defined as the difference between the threshold voltage and the most negative voltage within 5 ms of threshold, the voltages at 10 and 15 ms after the AP threshold, and the most negative voltage reached during any phase of afterhyperpolarization, for fast (fAHP), medium (mAHP), slow (sAHP), and maximum (maxAHP), respectively. Previous works have shown that the large-conductance (BK) and small-conductance (SK) calcium-activated potassium channels mediate the AHP following the AP up to 10 ms and up to hundreds of ms, respectively [39]. BK channels can influence the shape and frequency of the action potential to reduce the excitability of overactive neurons and enhance the excitability of less active neurons [40]. The timing of BK activation during the falling phase, relative activity of the cell, β subunit expression, and the type of other channels present in the membrane can contribute to the effect BK channels have on AP output [40]. BK channels require depolarization and calcium for activation and their calcium dependence is strongly tied to the membrane potential; an increase in calcium load results in an increased maximum AHP peak and a slower current decay [39, 41, 42]. SK channels are dependent upon increases in cytosolic calcium, generated during the AP, for activation [43, 44]. It has been shown by others that BK channels may contribute to either repolarization or AHP dependent upon expression of other voltage-activated currents [44]. Unlike BK channels, SK channels do not contribute to repolarization but have been shown to reduce AP threshold, stabilize the resting membrane potential, and limit firing of Aps [45]. Their activity contributes to a prolonged AHP during which the return to baseline also reflects the decay of intracellular calcium [44]. Considering that we found no significant effect of sex on threshold, AP half-width, overall maximum AP peak to peak firing frequency or maximum peak to peak frequency at any current step, and spike frequency adaptation during pre-adolescence, it is not likely that the reduced measures of AHP amplitude seen in pre-adolescent females are due to changes in SK activation. Rather it is likely a reflection of sex differences in BK channel contributions to AHP during this developmental stage.

In regard to mid-adolescent membrane properties, we observed a longer AP duration in D1R + MSNs from mid-adolescent male mice relative to females. The width of the mammalian action potential waveform is influenced by the activation of both voltage-gated sodium (Na_v_) and potassium (K_v_) channels [46]. While blocking Na_v_ channels has previously been shown to lengthen AP duration in MSNs in the NAc of adolescent rats, this manipulation also resulted in a reduction in AP amplitude [47]. As we found no evidence of sex differences in AP amplitude during pre- or mid-adolescence, our results suggest that rather than Na_v_ channels underlying the observed sex difference, K_v_ channels may be responsible. Activation of K_v_ channels limits cellular excitability by repolarizing the membrane after Na_v_ channels close [48]. Although there are many subunits of K_v_ channels expressed in the striatum, recent findings from Otuyemi et al. [49] indicate that, at least in the dorsal striatum, D1R + MSNs from adult mice of both sexes express K_v_2.1 and K_v_4.2 channels. The localization of K_v_2.1 (distributed across the soma and proximal dendrites) suggests that these channels may have contributed to our observed sex difference in AP waveform, rather than K_v_4.2 channels (which are on distal dendrites). K_v_2.1 channels may be found in non-conducting clusters or in conducting non-clusters, and in response to glutamate, clustered K_v_2.1 channels can disperse across the cell surface [49–52]. Therefore, it is plausible that during mid-adolescence, the number of and/or clustering patterns of K_v_2.1 changes over murine ontogeny, possibly in response to glutamatergic transmission, and that the ontogeny of K_v_2.1 is impacted by sex. Further evidence supporting this notion can be found in the work of Brundage and colleagues [53], who also observed evidence for sex differences in the function of K_v_ channels in the striatum of mice from PND 30 onward. In addition to Kv channels, BK channels can also impact repolarization and AP duration, at least in adult animals. However, in mid-adolescent rats (PND 38 to PND 42), blocking BK was shown to be ineffective in widening the AP of other cell types [54–56]. Furthermore, a change in BK channel activity would also be expected to alter AHP, and mid-adolescent animals in our study did not exhibit a sex difference in any measure of AHP. Thus, BK involvement in the different half-width is unlikely. Finally, we note that during mid-adolescence, half-width was not correlated with age in males but it was negatively correlated with age in females. During pre-adolescence, and on PND 35 (the beginning of the mid-adolescent age range), average half-widths were essentially identical between sexes, but overall, there was a significant sex difference in half-width for the mid-adolescent group. This indicates that the observed sex difference in half-width during mid-adolescence was driven by the older mice in this age range. Puberty in mice generally occurs 10–20 days after vaginal opening at about PND 26 in females and between PND 40–55 in males, but we did not specifically verify the onset of puberty in all mid-adolescent mice in our study, which ranged in age from PND 35–47 [57–60]. Therefore, we cannot exclude the possibility that the observed sex difference in half-width could be due to sex differences in the onset of puberty.

Regarding spontaneous excitatory postsynaptic currents (sEPSCs), we found a significant interaction of bin by sex in the cumulative probability distribution of interevent intervals for pre-adolescent D1R + MSNs. However, we did not observe a significant effect of sex on average sEPSC frequency or amplitude for either age group, indicating minimal sex differences in AMPA/kainate receptor-mediated spontaneous excitatory transmission. One caveat to these findings is that we only examined spontaneous events, which measures both action potential-dependent and -independent glutamate release. Future studies should also examine whether miniature EPSCs (action potential-independent events only) differ.

To our knowledge, only two groups, Lefevre and colleagues [61] and Willett and colleagues [22] have reported on excitatory synaptic transmission and membrane properties of NAcSh MSNs from both sexes, each finding no evidence of significant sex differences. Lefevre and colleagues conducted whole-cell recordings at PND 42–PND 63, an age range that spans portions of mid-adolescence and into early adulthood. While in the present study we also see no effect of sex in sEPSC amplitude or frequency in mid-adolescent animals, we do establish a sex difference in AP half-width, a measure that they did not examine. The second body of work, by Willett and colleagues, was conducted in rats at PND 21 in MSNs not identified by subtype, and the recorded neurons were in an area of the NAcSh that was more dorsal to that in our study. Thus, one interpretation of our finding of significant sex differences in the NAcSh during pre-adolescence, while Willett and colleagues did not find any at PND 21, could be that sex differences in membrane properties and cellular excitability have a species-specific ontogeny. On the other hand, the discrepancy in findings might also be a result of important methodological differences such as the medium spiny neuron subtype(s) targeted (D1R + only versus any MSN) or the area from which cells were selected for electrophysiology. Therefore, to better understand exactly when and where sex differences emerge, future studies should target specific populations of MSNs at multiple developmental stages and consider species as a potential variable.

## Perspectives and significance

Using both pre- and mid-adolescent, gonadally intact mice, we explored potential sex-based variations in membrane properties and excitatory synaptic transmission among dopamine D1 receptor-expressing medium spiny neurons in the nucleus accumbens shell. Our findings suggest potential sex differences at different developmental stages in glycine receptor and/or inwardly-rectifying potassium channel contributions to the resting membrane potential and input resistance of D1 receptor-expressing medium spiny neurons, BK channel modulation of action potential afterhyperpolarization, and potassium channel regulation of D1 receptor-expressing medium spiny neuron waveforms. Our results underscore the complexity of neural processes during adolescence and highlight the significance of sex-specific considerations in the functioning of the shell of the nucleus accumbens. Future research is essential to pinpoint the precise molecular mechanisms involved, offering potential targets for future studies investigating disorders or conditions with prevalence and symptomology that differ by sex and stage of development.

## Data Availability

The datasets used and analyzed during the current study are available from the corresponding author on reasonable request.
